# Deutche Monatschrift Fur Zahnheilkunde

**Published:** 1886-03

**Authors:** Jul. Parreidt

**Affiliations:** Leipzig


### Deutche Monatschrift fur Zahnheilkunde.

The editorial address with which Herr Julius Parreidt opens the January number of the *Deutsche Monatschrift fur Zahnheilkunde*, contains a useful history of the contents of the first twenty-five volumes of this periodical, and many excellent remarks on professional journalism We give a translation for the benefit of the readers of the REGISTER.

With the preceding number the journal closed its twenty-fifth volume as the organ of the Central Society of German Dentists, with the present it enters on the second quarter century of its existence. It may not be improper on such an occasion to give a sketch of the history of our journal, to see what it has done, and what duties remain to be accomplished.

To repeat the words of the preface of the first volume (1861), the publication of a German dental journal was the first fruit of the Central Society of German Dentists, founded in 1859. The necessity of entering into a more intimate communication, and of recounting and explaining the results of experiment and practice called for the foundation of the Central Society of German Dentists, and a necessary consequence was the creation of a society journal, not only as the organ of the said society, but as the organ of the whole dental profession of the country, the serving of whose necessities, and the protection of whose interests would be its especial duty.

The tendencies of the journal would be those of the society since it would always preserve a strictly scientific mode of thought by the insertion of important original papers, on all subjects relating to dental art and science, and by the collection of all the latest and most scientific articles having any bearing on dentistry. And in conclusion the same preface states that it is above all important to create an organ which shall worthily represent the German profession of dentistry amongst foreigners.

This programme was adhered to not only by Heider, the first editor, but by his successors. From the first to the twenty-fifth volumn we find a continued succession of excellent orginal papers on the different departments of daily practice and essays of a very scientific character.

Heider died in 1866. His successor was Zur Nedden, how had been associated in the editorial work from the commencement, and had contributed many highly interesting and important articles on different dental subjects, specially on the branch of education, and had moreover selected the abstracts from other journals.

At the end of the 10th year Zur Nedden resigned the editorship, and from 1871 to 1872, Muhlreiter occupied the post temporarily. He would not, however, definitely accept the editorship. On his resignation the organ, which up to that time had been published by the Society itself (at first through Carl Gerold's son, in Berlin, and from 1868, through J. A. Stein, in Nuremburg,) was placed for publication in the hands of Arthur Felix, of Leipzig, who thereupon discontinued his monthly journal, the *Zahnarzt*, which had appeared regularly from 1846 to 1872. The price of the *Vierteljahrschrift*, which was first 5 than 8 marks, was now increased to 12 marks annually. Zeitmann was unanimously chosen Muhlreiter's successor, he was, however, prevented by illness from performing the duties of the office, and Dr. Klare, the President of the Central Society, appointed as editor, Robert Baume, one of the contributors to the journal, but not at that time member of the society, he had, however, made a reputation by his papers "On the behavior of the pulp in certain pathologic condition of the alveolar process, etc.

The new publisher issued the Society's organ in a more elegant form, but there seems to have been considerable difficulty in obtaining fitting matter to fill the journal which had been enlarged from 20 to 30 sheets. In consequence of this the editor was himself compelled to contribute many excellent essays. After these changes the organ of the society entered on a quietly successful career.

Since 1876 the wish had been expressed for a monthly instead of quarterly publication, but the contributions were hardly yet sufficiently numerous. In the 22d vol. the number of sheets was increased to 32½, and the subscription raised to 13 marks.

From 1883 the journal appeared monthly without any promise of an increase in annual contents. In consequence of the augmented postal and express charges for a monthly publication the subscription was raised to 14 marks, but reduced to 10 marks for members of the society. Owing to the industry of the contributors and the increase of sales, the normal 32 sheets of the complete volume increased to 33 in 1883, 35 in 1884, and reached 37 in 1885.

After Baume had held the editorship twelve years he resigned his office in the twenty-fourth year of the publication, and the undersigned succeeded him. I undertook the editorship in the hope of working up to the programme published by Heider in 1861, and adhered to by his successors, and of thereby being of service to the dental profession.

It is impossible in a mere sketch to do more than name some of the contributors of the many valuable papers contained in the twenty-five volumes of the *Vereinsorgan.* In the departments of microscopic and pathologic anatomy, I note specially Wedl, Heider, Hohl, Muhlreiter, Baume, Schlenker, and recently Walkhoff and Morganstern.

The following have written on practical matters, clinic, operations, materia medica, etc., Niemeyer, Tofoha, Hering, Klare, Zur Nedden, v. Langsdorff, Steinberger, Hartung, Scheff, Kleinmann, Sauer, Witzel, Detzner, Schlenker, Scheller, Hollaender, Schneider, Schreiter, Wellauer, Hagelberg, Telschow, Skogsborg, Kuhns, Kahndt, Schwartzkopff and many others.

From 1868 onwards have appeared numerous papers and discussions on nitrous oxide, specially those of Sauer, Grohnwald, Telschow and Blumm.

On prosthetic dentistry and allied subjects, the following have contributed, specially to the earlier volumes, many papers, viz: Von Humm, Weber, Niemeyer Klare, Blume and Muhlreiter. The discussion on the value of rubber for plates has frequently been revived. After the wars of 1866 and 1870 appeared papers on the rubber splint for fractures of the jaw by Haun and Suerson, and a few years since Sauer wrote on the wire splint. The method of Schrottsky for obtaining impressions, led to the development by Suerson, of a system of obdurators, and finally to that of a rational obdurator, which has been more recently modified by Schiltsky Hartung, Kleinmann, Sauer and Witzel have also written on obturators and maxillary prosthesis.

We must not omitit to name the contributionss on aluminum which have appeared since 1864, especially those of Sauer in 1871, the essays and discussions on celluloid which commenced in 1870, and the accounts of the many experiments on continuous gum work by Herbst, Thockow, Schiltsky, etc.

The valuable extracts and translations were furnished for ten years by Zur Nedden, and after that, amongst others, by Von Langsdorff Petermann, Schneider, Koconthal, and latterly by Schwartz Kopff.

Zur Nedden and Heider have contributed articles of unusual interest on the question of education. A consideration of the incisive, impressive and logical statements in favor of scientific and practical education made by Zur Nedden, between 1861 and 1869, in connection with the later utterances of Von Langsdorff and others in the same direction, and the fact that now in our days the aim of their efforts has only been partly reached, will show the difficulties of obtaining from governments any assistance in the interests of a special profession.

What is the future task of the *Vereinsorgan*? Simply, I think, to follow the path already chosen, keeping in view continually the interests of true science and the elevation of the dental profession.

Of the original articles which appear in the *Monatschift*, those have the highest and most lasting value, which contain careful observations and experiments. I had the good fortune when I I first became editor, to be amply supplied with good material which continued to come in, in such quantity that I was unable to publish it as rapidly as desirable. It will be possible this year to publish both original contributions and selections with greater punctuality.

In the preface to the twenty-fifth volumn I stated that articles on the social position in relation to the elevation of the profession should appear.

The earlier efforts of the society for the elevation of the profession by better technical training having recently received partial satisfaction in the formation of the dental institutes in Berlin and Leipzig, a feeling of repose has affected this department of literary activity. In the last few months there has been a revival of interest in it.

There are still other ways and means for the elevation of the profession, and it is to be hoped that anything new in this direction will be discussed either personally in the meetings of the society, or in the columns of the *Monatschrift*.

The Central Society of German Dentists resolved at its last annual meeting by increasing the number of pages in the journal, to allot more room than formerly to the discussion of professional interests without encroaching on that devoted to scientific articles. It must be clearly understood (to avoid former misunderstandings on this point,) that contributions on professional questions, as in the case of all others must be entirely original, and must treat of the matter from some new stand-point. It is to be hoped, however, that the activity of both central and local societies will not be diverted from those strictly scientific questions, which are largely the interest of the profession. In order to prevent this eventuality, it has been agreed between the publisher, the editor, and the president of the society to arrange the original articles, selections and proceedings of societies within the usual number of pages, and that the surplus matter, more especially that treating on questions of professional standing shall be published in a supplement.

In conformity with Heider's programme by which the Vereins organ has been successfully edited for twenty-five years, we shall endeavor to maintain the strictly scientific character of the *Monatschrift,* in order that the organ of the Central Society of German Dentists may bear witness before all the world to the skill of German dentists.

JUL. PARREIDT.

LEIPZIG, December, 1885.

(Translated by William Beer.)

